# Automated machine learning recognition to diagnose flood resilience of railway switches and crossings

**DOI:** 10.1038/s41598-023-29292-7

**Published:** 2023-02-06

**Authors:** Jessada Sresakoolchai, Mehmet Hamarat, Sakdirat Kaewunruen

**Affiliations:** Department of Civil Engineering, University of Birmingham, Birmingham, B15 2TT UK

**Keywords:** Natural hazards, Civil engineering, Mechanical engineering

## Abstract

The increase in demand for railway transportation results in a significant need for higher train axle load and faster speed. Weak and sensitive trackforms such as railway switches and crossings (or called ‘turnout’) can suffer from such an increase in either axle loads or speeds. Moreover, railway turnout supports can deteriorate from other incidences due to extreme weather such as floods which undermine cohesion between ballast leading to ballast washaway or loss of support under turnout structures. In this study, new intelligent automation based on machine learning pattern recognition has been built to detect and predict the deterioration of railway turnouts exposed to flooding conditions which is the scope of this study. Since the turnout system is very complex by nature, different features and smart filtering are explored to find the potential features for deep learning. Nonlinear finite element models validated by actual field measurements are used to mimic the dynamic behaviors of turnout supports under flooding conditions. The study exhibits that the novel recognition model can achieve more than 98% accuracy, yielding the potential capability to recognize and classify turnout support deteriorations facing extreme weather conditions which will be beneficial for responsible parties to schedule and plan maintenance activities.

## Introduction

Railway infrastructure comprises different components to make it able to serve railway transportation purposes. This study will focus on railway turnouts which are one of the most significant parts of the railway system. Turnouts are sets of components of mechanical equipment that steer rolling stocks from one track to another track. For this function, railway turnouts have to be installed at railway junctions, railway spurs, siding tracks, or branches where rolling stocks need to change directions. From this, turnouts are movable railway parts which used to define the directions of rolling stocks. One set of a turnout also contains many components such as pairs of switch rails, point blades that lie between the stock rails, crossing noses, connection parts, frogs, or guard rails. The movement of these parts is lateral. In normal cases, turnouts with the full function will be locked after the movements have been done to define the directions of rolling stocks for smooth operations and safety purposes. It can be seen that, due to their functions, turnouts are applied by high loads and impacts regularly during the operation^1–3^ and this is a reason why turnouts are one of the weak points in the railway infrastructure^4–9^. Due to the high loads and impacts, turnouts tend to deteriorate relatively fast. In addition, turnout supports are also affected by high loads and impacts. After all, forces are transferred from the railway infrastructure to the supports underneath because the forces are distributed along with the support layers. For example, ballast used to support rails and sleepers deteriorates due to regular operations. Therefore, not only do railway turnouts deteriorate but also their supports deteriorate. Besides the regular operations, turnout support can be accelerated the deterioration of some extreme events such as flooding events.

From previous studies, flooding events were shown to be the most prevalent extreme events. In addition, they have caused damage to railway systems all around the world^10^. In 2003, the Rail Safety and Standards Board (RSSB) publishes a report stating that there have been 129 flood-related incidents^10^. This is because flooding can cause damage to railway structures such as bridges, track supports, turnout supports, or electrical and mechanical systems.

Although railway turnouts are not damaged by flooding, they can be damaged by regular operations. Each time of damage can disturb railway operations in terms of delays, damages, or even derailment^11^. Because turnouts are sensitive components in the railway infrastructure, railway operators tend to prioritize their maintenance of them. Xin et al.^4^ reported that over 400 turnouts must be replaced every year. Moreover, two of them are required immediate repair every week. The cost of the turnout replacement in the Netherlands is around 6.4 million euros each year. From this, it can be concluded that railway turnouts are railway components that must be controlled and highly prioritized as well as their supports.

**Figure 1:**
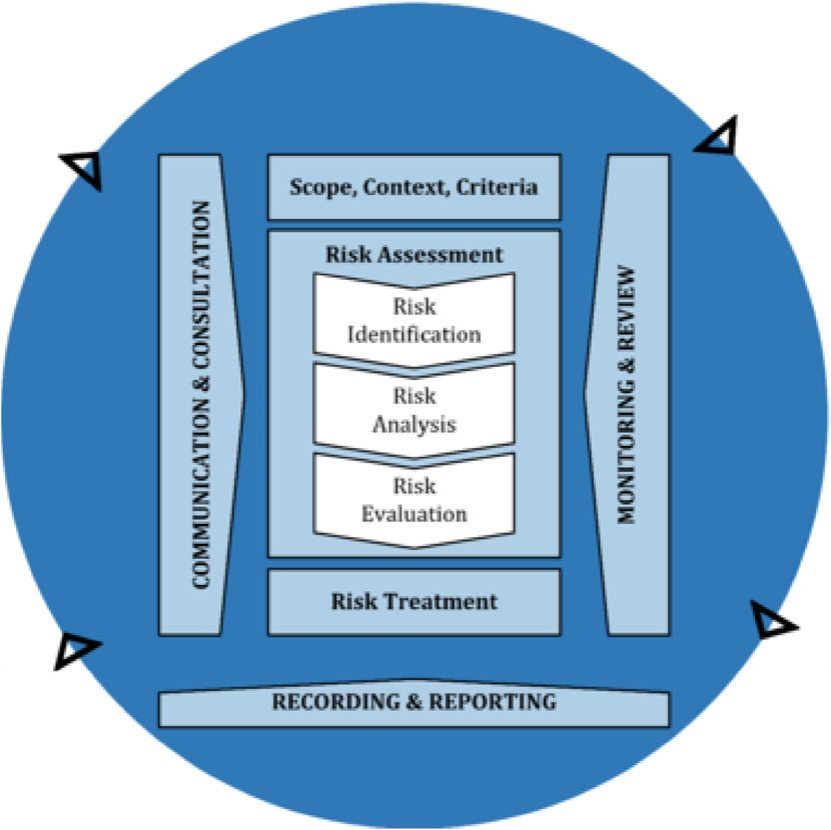
Risk management process^12^.

This study focuses on predicting and detecting the deterioration of turnout supports. The objective is to develop machine learning models that can anticipate or estimate the deterioration of turnout supports utilizing support stiffness as a key signal. Axle box accelerations (ABAs) or vibrations from the front wheels of rolling stocks as well as crossing noses displacements are used as features of the machine learning models. The use of ABAs or crossing nose displacement to detect and predict the turnout support deterioration seems to have the potential to provide a high accuracy when they are integrated with machine learning. Previous studies which will be discussed in the literature review normally used only the maximum or average values of ABAs, displacement, and other values. However, use of the machine learning can detect a hidden pattern that humans cannot recognize and use them to make a prediction with high accuracy. Moreover, the cost of installation is not high because only a few sensors are required to be installed and they are not complicated. The study’s expected contribution is that the developed machine learning models will be able to detect and predict the deterioration of turnout supports which will be useful for railway maintenance planning and management. The developed approach can be used to support decision-making for railway maintenance. At the same time, it can reduce the risk of failure because the deterioration can be monitored regularly based on regular operations that are used to collect data. The detection can provide insight of the current condition of the track structure in advance to avoid critical damages that cause relatively high maintenance costs. In addition, this study applies ABAs to predict so only accelerometers are required to install which is cheap and simple. The measurement can be done with regular operations without disruption. Therefore, this approach will integrate operation and maintenance seamlessly which improves the cost-efficiency and smoothness of the railway system. The proposed approach can be used to screen the support condition in no time. If the monitoring needs to be more precise, specific tools can be allocated after the screening presents results that reach the threshold. An advantage of using ABA from regular operations using only service trains is biased because the operations are very limited in terms of speeds, stops, and timetable. This will negatively affect the diversity of data which is not desirable. In this stage, the study will not include the probability of failure because it requires performance and action functions when the scope of this study focuses on the performance function, which relates to asset operations. Based on ISO 31,000:2018 (Risk management—Guidelines)^12^, this study tries to develop a tool for risk monitoring or to detect the condition of the support. After that, further processes of risk management can be done according to the standard such as risk assessment and risk treatment. Different railway authorities apply this approach to manage risks and uncertainty. Especially for uncertainties, railway authorities rely on inspections and condition monitoring to aid asset operations (Fig. 1).

## Literature review

As mentioned, flooding events are one of the most common extreme events in the railway industry. However, they are not prioritized as the most significant ones^10^. However, the railway system must be capable of effectively managing floods. That means not every flooding occurrence may be avoided. In order to handle railway preventative maintenance, risk management is the critical approach used to deal with these incidents. Because flooding cannot always be avoided, being able to detect and predict the damage it does will be useful for railway maintenance. Hasnayn et al.^13^ investigated the performance of the railway subgrade during and after floods. They discovered that flooding had a considerable influence on track stiffness because of settlement and water content that changed from the desirable conditions. They also proposed that following floods, railway support should be thoroughly studied.

Hamarat et al.^14^ explored the dynamic behavior of railway turnout supports in the case of floods. They conducted the study using a finite element method (FEM). From their study, flooding had an important role in the deterioration of track geometry and railway defects. some of their findings were that the superelevation could not be maintained at the same level as the design after flooding and both short twists and long twists twist tended to be extremely poorer after the flooding incidents. It can be seen that flooding tends to accelerate the deterioration of track supports. Therefore, in case of flooding, the strengths of turnout supports have to be investigated although the incidents are not severe or the water levels are not high.

To be able to detect and predict the deterioration of turnout supports, there are different parameters that can be used as indicators. Li and Berggren^15^ discovered that global track stiffness was related to the track’s performance. In that study, they considered the dynamic responses as the representatives of the track performance. They applied both static and dynamic methods to study the relationships. They stated that railway and turnout support stiffness were directly related to dynamic responses such as sleeper accelerations, wheel-rail forces, or rail moments. Their findings can be applied to this study. To identify the turnout support deterioration, this study will use support stiffness as the indicator used to measure the turnout support deterioration.

To measure the stiffness of track supports, the current techniques can be categorized into two groups, on-board approaches and track-side approaches. Examples of the onboard approaches are the application of the Swiss track stiffness measuring vehicle, the rolling stiffness measurement vehicle of the Swedish railway^16^, or other measurement vehicles which are also used in different countries such as China and the USA. The main disadvantage of using onboard approaches is that special vehicles are required to measure the interesting parameters so the capital cost and operation cost are relatively high. Moreover, the running of these measurement vehicles can also disrupt regular operations if the speed of measurement vehicles is not equal to the speed of regular operations. In addition, due to the high cost, the measurement and inspection cannot be done frequently so severe deteriorations can be overlooked in some cases. Another limitation of the onboard approach is low accuracy^17^. For the track-side approaches, examples of them are track deflection techniques, cameras, accelerometers^18^, laser arrays, and geophones^19^. Like the on-board approaches, the track-side approaches also have disadvantages. Examples are the high cost of installed equipment and sensor including software that have to be purchased. This study aims to propose alternative approaches to inspect the track or turnout support stiffness to overcome these limitations while the accuracy of the detection is acceptably high. The authors obtain some ideas from both on-board and track-side approaches. This study proposes to use axle box accelerations (ABAs) and crossing nose displacement to detect and predict railway turnout support deterioration. It has been proven that the use of ABAs provides a satisfying outcome while the crossing noses are the weak point in the railway turnout structure^4^ so this location can be used to detect and predict deterioration. These techniques tend to be more interesting in the present because they are relatively cheap, do not disrupt the regular operations because the measurement can be done together with the regular operations, they are fast because the measurement is done at the same speed as the regular operations, and only a few sensors need to be installed. However, the use of only ABAs and crossing nose displacement might not provide a satisfying performance if only maximum values or average values are used because different defects can result in the same maximum and average values. Moreover, the load and speed of rolling stock also affect the maximum and average values of both ABAs and crossing nose displacement. A potential approach to make this concept comes true is the use of machine learning when it is used to do the prediction using raw data with the benefit of machine learning to detect patterns hidden in the data.

Kaewunruen^20^ used the dynamic wheel and rail interaction to estimate the structural deterioration of railway turnouts. They used the average maximum accelerations to identify the deterioration of turnouts. This finding is also support by Cao et al.^21^. Sysyn et al.^22^ applied the machine learning concept to develop a model to detect railway turnout defects. They developed the binary-class machine learning model or the developed model could classify only whether the interesting section had defects. In their study, ABAs were used as the feature to develop the machine learning model. The highest accuracy that they could achieve was 90%.

Other examples of the use of ABAs in the railway maintenance purpose are In 2021, the use of ABAs to detect and classify the severity of wheelflats^23^, to detect and classify the railway combined defects^24^, different railway defect detection^25–28^, defect severity classification^29–33^ or railway operation^34,35^. Moreover, machine learning also has the potential in different areas such as geoengineering and geoscience^36–39^.

From the literature review, the ability to detect and predict the turnout support deterioration is critical, especially in the case of flooding and although different approaches seem to have some limitations, the machine learning approach tends to be able to overcome those limitations while the delivered accuracy is still acceptable, especially machine learning modes that have an ability of pattern recognition such as convolutional neural network (CNN). In addition, from the literature review, there has not been any previous study studying railway switches and crossings under flooding conditions applying machine learning. Therefore, this study aims to explore the potential of the use of a machine learning approach to detect and predict railway turnout support deterioration through support stiffness when ABAs and crossing nose displacement are used as the key features to develop the machine learning models.

## Methodology

### Finite element model development and validation

**Figure 2:**
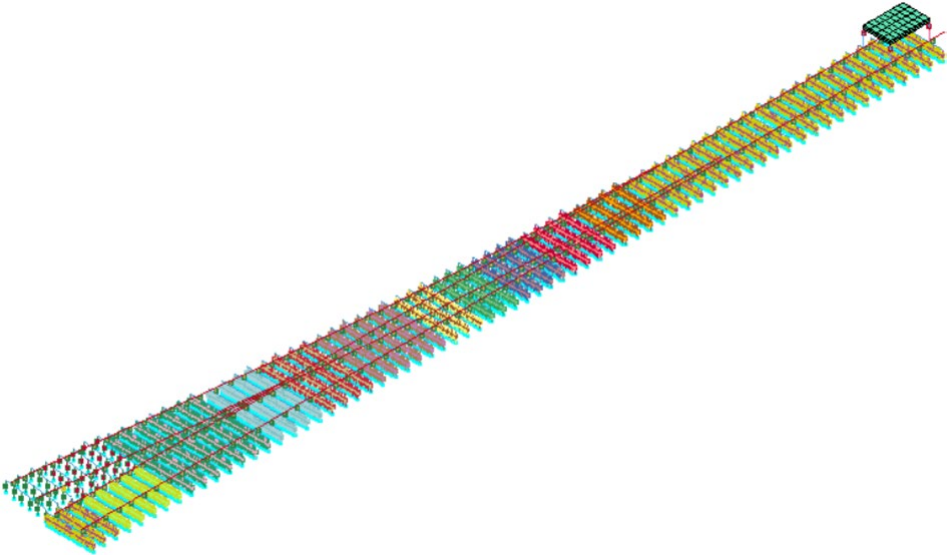
The 3D vehicle-slab FEM model^40^.

This study uses the FEM concept to develop a nonlinear FEM model to mimic flooding events. In addition, using the FEM model can also enrich the data sets in diverse situations which is suitable for machine learning model development. It is worth noting that it can remove a critical negative characteristic of data which is biases due to the data diversity. In some cases, data with high biases can yield nearly 100% accuracy which is undesirable in the machine learning field. With data diversity, machine learning will be capable of dealing with situations outside normal service conditions. The nonlinear FEM models are developed based on the model developed by Hamarat et al.^40^. The FEM models are developed as the 3D vehicle-slab model which is shown in Fig. 2. In the models, rolling stocks are modeled using the concept of multi-body simulations. The FEM models are developed using LS-DYNA software which is a popular FEM software.

In the FEM models, railway turnouts are modeled using a 1:9 crossing angle turnout as a case study. The rolling stock models consist of a car body, two bogies, four wheelsets, primary suspension, and secondary suspension. The ballast track models consist of rail, rail pads, sleepers, and ballast. Rails are modeled using the Euler beam concept. The rails are supported by rail pads which are imitated by using a series of springs and dampers. After that, rail pads are supported by sleepers which are also modeled using the Euler beam concept. Under the sleepers, the ballast bears the sleepers and it is modeled using the springs and dampers to imitate the behaviors. S01-SPRING_ELASTIC and S02-DAMPER_VISCOUS are the LS-DYNA keywords that are used to imitate the series of the stiffness and damping for rail pads and ballast.

The vertical wheel-rail contact stiffness is also changed to mimic the real-world situation in which the track stiffness is not consistent. In LS-DYNA, the built-in keywords *Rail_Track and *Rail_Train are used to model the interaction between the wheel and the rail. To mimic the dynamic behavior of rolling stock and railway structure as much as possible, different bounties in LS-DYNA are defined to limit movements in some directions of the railway infrastructure such as the bottom of the ballast which the boundary is set to have non-vertical displacement or the ends of rails that cannot have the displacement in any directions.

The developed FEM models are verified with the filed measurement data which is open-source^41,42^. The field measurement consists of the measurement of accelerations. This conforms to the interesting outputs from FEM models that will be used in this study because ABAs are forms of accelerations that are measured by using accelerometers. To be able to compare the outputs from the developed FEM models and the field measurement data, parameters in the FEM models are set to be the same as the field measurement by Wan et al.^41^. The maximum accelerations from the developed FEM models and the field measurement data are 201.6 m/s^2^ and 214.05 m/s^2^ respectively. It can be seen that the difference is about 5% which is acceptable. Therefore, it can be concluded that the developed FEM models can be used to generate numerical data.

**Figure 3:**
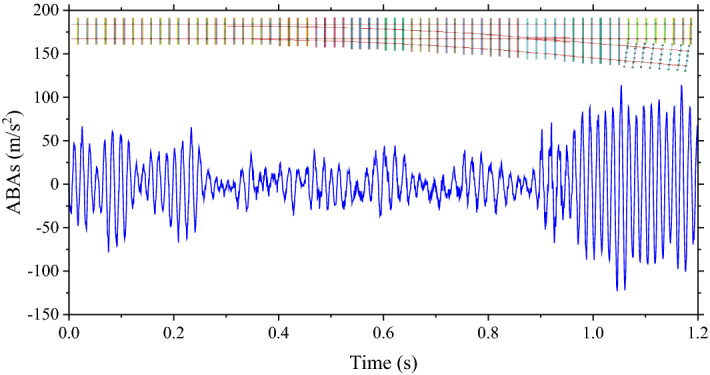
Example of ABAs.

**Figure 4:**
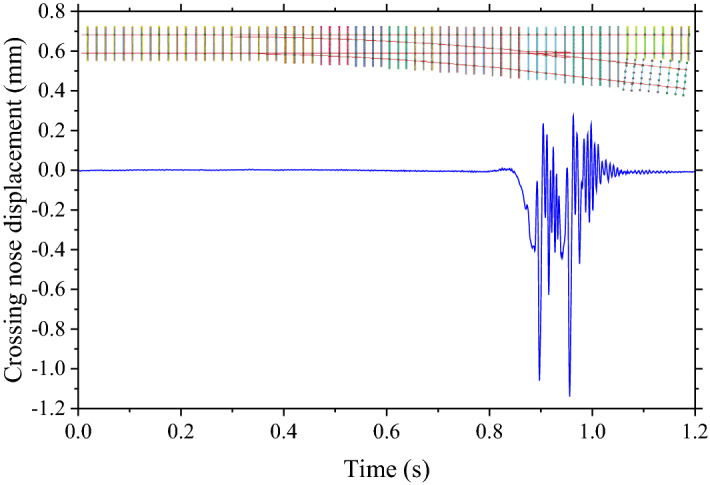
Example of crossing nose displacement.

To mimic the railway turnout support deterioration under flooding events, ballast stiffness and damping coefficients are modified depending on water levels^14^. Table 1 shows the range of variance based on water levels ranging from 0–114%^14^. Please be noted that the stiffness of the interesting section is not the same for the whole section. This study will use the stiffness at the turnout support as the key indicator. The stiffness of every part in the section is varied based on the water level as mentioned. In addition, various parameters such as rolling stock weights and speeds are modified to provide data variance. Table 1 includes these as well. In addition, Table 1 includes the class for the classification of the machine learning model. The classes for the prediction are separated based on the turnout support deterioration or stiffness and damping coefficient as shown in the table. As mentioned, this study will use ABAs and crossing nose displacement are featured to train the machine learning models. Examples of outputs from the FEM models are shown in Figs. 3 and 4 respectively. Both of them are outputs from the simulation when the speed of the rolling stock is 135 km/h, the weight of the rolling stock is 46.4 tons, the stiffness of the turnout support is 14.6 MN/m and the damping coefficient of the turnout support is 1.3 kNs/m. From Fig. 2. , it can be seen that the location of the turnout is about the end of the section. Therefore, the ABAs tend to be high at the end of the simulation as shown in Fig. 3. At the same time, from Fig. 4, the displacement of the crossing nose is 0 until the rolling stock passes through it at about the end of the simulation. Then, after the rolling stock completely passes, the displacement is back to 0 again.

**Table 1:** Ballast stiffness and damping coefficient variations.

Parameters	Units	Ranges	Class
Stiffness	MN/m	More than 12.8	1
		10.5–12.8	2
		8.1–10.5	3
		Less than 8.1	4
Damping coefficient	kNs/m	Less than 1.2	1
		1.2–1.5	2
		1.5–2.0	3
		More than 2.0	4
Weights of rolling stocks	Tons	32–48	n/a
Speeds of rolling stocks	Km/h	135–225	n/a

### Data preparation and machine learning model development

There are 1936 simulations in total. As mentioned, this study applies the FEM method to generate the numerical data. Outputs exported from simulations are used to train the machine learning models. The outputs are used in the form of raw data or time-series data of ABAs from two front wheelsets and/or displacement of the crossing nose. Both outputs from simulations are used together and separately to explore the performance of the machine learning models. Stiffness is used as the key indicator to identify the conditions of railway turnout supports. Irregularities of tracks are included in the finite element model. Not only reflecting the real condition of the tracks, but irregularities also represent uncertainty in the railway system’s nature. To train the machine learning model, there are three forms of features; (1) two sets of time-series data from ABAs, (2) time-series data from crossing nose displacement, and (3) the combination of (1) and (2). The outputs from FEM models are different in shape based on the speed of rolling stock when the length of the experimental section is approximately 45 m.

A convolutional neural network (CNN) is a machine learning technique that is utilized to create the predictive model in this study. Data in its raw form are supplied into the CNN model. This machine learning technique is chosen because it is a powerful deep machine learning technique that can be utilized to tackle a wide range of problems and it is proven that the performance of the model is satisfying. CNN is a common pattern recognition algorithm that is appropriate for the difficulties in this study. Two outstanding properties of the CNN model are it can extract significant features from inputs via the feature extraction part in the model’s architecture. In other words, this ability is called smart filtering which makes CNN outstanding in terms of pattern recognition.

The machine learning models are developed based on two problems in this study. The first one is the classification when the classes of predictions are referred to in Table 1. The railway turnout support deterioration is classified as a class when the ranges of stiffness are shown in the table. From the table, there are four classes according to the severity of the deterioration. The second one is the regression model when the machine learning model is developed to predict the current stiffness of railway turnout supports.

70% of the data are utilized to train the machine learning model, while the remaining 30% are used to test the model. In the data splitting process, the proportions of each class are maintained the same in both training data and testing data using stratified sampling. From Table 1, there are four classes. It is initially planned that each class will have a proportion of about 25% so this proportion will be maintained in both training data and testing data. To ensure that each model produces the best possible performance, hyperparameter tuning via grid search is applied. Hyperparameter tuning is used to improve the performance of the machine learning model because not every parameter in the machine learning architecture is tuned during the training process. The parameters that are tuned are weights and biases while other hyperparameters such as the number of nodes, filters, or layers are fixed. Therefore, hyperparameter tuning has an important role to explore the optimized combination of hyperparameters providing the best performance. The concept of grid search is the machine learning model will try every combination that is defined by the grid search. For example, if two hyperparameters are aimed to be tuned and there are three values to be tried for each, the total number of combinations is nine. To ensure the performance of the machine learning model, the range of each hyperparameter has to be comprehensive and based on the characteristic of the data. In the study, the hyperparameter tuning is performed using the loop creation in the code during the machine learning development stage. In each loop, one hyperparameter is defined within the selected range. The number of the loop will be equal to the number of hyperparameters that are tuned. Then, the indicators used to measure the performances are recorded using an array. After that, the performance of each hypermeter combination is compared to find the combination providing the best performance. Table 2 presents the list of the tuned hyperparameters for the CNN model.

**Table 2:** List of hyperparameter tuning.

Hyperparameters	
Number of convolutional layers	Activation function
Filter	Batch size
Kernel size	Optimizer
Number of pooling layers	Number of hidden layers
Dropout	Number of hidden nodes
Pool size	

The novelty of the developed machine learning model is the machine learning model in this study uses raw data or time-series data that previous studies have never done before. Normally, defect detection in the railway system uses only the maximum value of the accelerations or displacement only. On the other hand, this study tries to create novelties and dominate the state of the art’s performance by using raw data because this study aims to take the benefit of the CNN model that contains the feature extraction part. In other words, previous studies tend to use maximum and/or average values as inputs together with the speed or weight of rolling stock. By using this, machine learning model training can be misleading because other incidents such as irregularities can also cause the same value with target deterioration. The feature extract part will search for insight into the data and pattern to do a prediction without the requirement of feature engineering. Therefore, the selection of features is not required to be done by humans but the machine will discover itself and there is no loss in the data processing process. In addition, this study stacks raw data as layers. for example, if the features are ABAs, the shape or dimension of the features will be [2 × 1 × timestep]. The first number (two) is the number of feature layers which is two consisting of the first and second sets of ABAs. One means the number of rows of features which is one representing 1-d time series data. The last number of the shape is the number of columns or timesteps which represents the ABAs or crossing nose displacement. If the features are crossing nose displacement, the number of layers of feature is only one because there is only one set of data. At the same time, if both ABAs and crossing nose displacement, the number of layers will be three consisting of two ABAs and one crossing nose displacement. The cost functions used to train machine learning models are depended on the type of problems. Accuracy is used for the classification and root mean square error (RMSE) is used for the regression.

To assess the performance of the machine learning models that have been developed, different indicators are used based on the characteristics of the problems. The indicators used to evaluate the performance of the classification model are accuracy, precision, and recall. The indicators that are used to evaluate the performance of the regression model are mean absolute error (MAE), root mean square error (RMSE), R^2^, mean percentage error (MPE), precision, recall, and accuracy. Equations used to calculate each indicator are shown in Eq. 1-Eq. 7 where ${y}_{i}$ is a true value, ${x}_{i}$ is a predicted value, $n$ is the number of samples, $\overline{y }$ is the mean of true values, $TP$ is a true positive value, $FP$ is a false positive value, $TN$ is a true negative value, and $FN$ is a false nagetive value. From the mention indicators, the lower values of MAE, RMSE, and MPE represent the better performance of the models while the higher values of R^2^, precision, recall, and accuracy represent the better performance of the models. It is to be noted that the highest possible values of R^2^, precision, recall, and accuracy are 1.00 which means the models can predict every value correctly.

1 $$ MAE = \mathop \sum \limits_{i = 1}^{n} \left| {\frac{{y_{i} - x_{i} }}{n}} \right| $$

2 $$ RMSE = \sqrt {\frac{{\mathop \sum \nolimits_{i = 1}^{n} \left( {y_{i} - x_{i} } \right)^{2} }}{n}} $$

3 $$ R^{2} = 1 - \frac{{\mathop \sum \nolimits_{i = 1}^{n} \left( {y_{i} - x_{i} } \right)^{2} }}{{\mathop \sum \nolimits_{i = 1}^{n} \left( {y_{i} - \overline{y}} \right)^{2} }} $$

4 $$ MPE = \frac{1}{n}\mathop \sum \limits_{i = 1}^{n} \left| {\frac{{y_{i} - x_{i} }}{{y_{i} }}} \right| $$

5 $$ Precision = \frac{\sum TP}{{\sum \left( {TP + FP} \right)}} $$

6 $$ Recall = \frac{\sum TP}{{\sum \left( {TP + FN} \right)}} $$

7 $$ Accuracy = \frac{{\sum \left( {TP + TN} \right)}}{{\sum \left( {TP + FP + TN + FN} \right)}} $$

## Results and discussion

From the machine learning model development, the performances of the models according to the characteristics of problems are shown in Section “Model performances” and the set of optimized hyperparameters is shown in Section “An optimal set of hyperparameters”.

### Model performances

#### Classification model

The severity of the turnout support deterioration is shown in Table 1. There are four classes as shown in the table ranging from class 1 to 4. Class 1 represents the best condition of the turnout support while class 4 represents the worst condition of the turnout support. As mentioned, this study explores the potential of the use of features in three different formats, two time-series data of ABAs, displacement of crossing noses, and the combination of both. The performances of the classification model are shown as confusion matrixes and model reports in Tables 3, 4 and 5.

**Table 3:** Classification model’s performance when features are ABAs.

	Pred. class 1	Pred. class 2	Pred. class 3	Pred. class 4	Recall
True class 1	130	0	1	1	0.98
True class 2	8	125	7	0	0.89
True class 3	0	0	160	0	1.00
True class 4	0	0	0	149	1.00
Precision	0.94	1.00	0.95	0.99	0.97

**Table 4:** Classification model’s performance when features are crossing nose displacement.

	Pred. class 1	Pred. class 2	Pred. class 3	Pred. class 4	Recall
True class 1	142	7	0	0	0.95
True class 2	14	133	1	0	0.90
True class 3	0	13	131	2	0.90
True class 4	0	0	2	136	0.99
Precision	0.91	0.87	0.98	0.99	0.93

**Table 5:** Classification model’s performance when features are ABAs and crossing nose displacement.

	Pred. class 1	Pred. class 2	Pred. class 3	Pred. class 4	Recall
True class 1	127	2	1	0	0.98
True class 2	0	143	3	0	0.98
True class 3	1	1	148	0	0.99
True class 4	0	0	1	154	0.99
Precision	0.99	0.98	0.97	1.00	0.98

From the above tables, it can be seen that the use of both ABAs and displacements of the crossing nose provides the best performance followed by the use of ABAs. The best accuracy that the model can achieve is 0.98 which is satisfyingly high. However, if the installation is too high for some situations, the option to install only one sensor also provides acceptable accuracy. From the model trained with the crossing nose’s displacement, the accuracy is 0.93 which is good enough to support the decision-making in railway maintenance and it requires only one sensor to install at the crossing nose. The sensor can be laser-based or a camera to track the movement of the crossing nose. On the other hand, the installation of accelerometers at axle boxes of rolling stocks provides more accuracy. In addition, data from accelerometers (ABAs) can be used to detect other defects when rolling stocks are operated. However, the use of any approaches presented in this study can provide a satisfying performance of the detection and prediction. When considering precisions and recalls, the outcomes are accorded to the accuracy that the use of both ABAs and crossing nose’s displacement tends to provide the best values. It can be seen that, when both features are used, precisions and recalls of all classes are higher than 0.97. That means the developed machine learning model can detect the turnout support deterioration accurately referred to high recall. At the same time, the predictions of every class are highly reliable referring to the high precision.

#### Regression model

As mentioned in Section “Data preparation and machine learning model development”, the indicators used to identify the performance of the regression model are MAE, RMSE, R^2^, and MPE. The comparable parameters are turnout support stiffness. The performances of the regression models are shown in Table 6.

**Table 6:** Regression model’s performance.

Indicators	ABAs	Crossing nose’s displacement	ABAs and crossing nose’s displacement
MAE	0.28 MN/m	1.08 MN/m	0.21 MN/m
RMSE	0.36 MN/m	1.45 MN/m	0.30 MN/m
R^2^	0.98	0.70	0.99
MPE	2.54%	11.56%	1.95%

**Figure 5:**
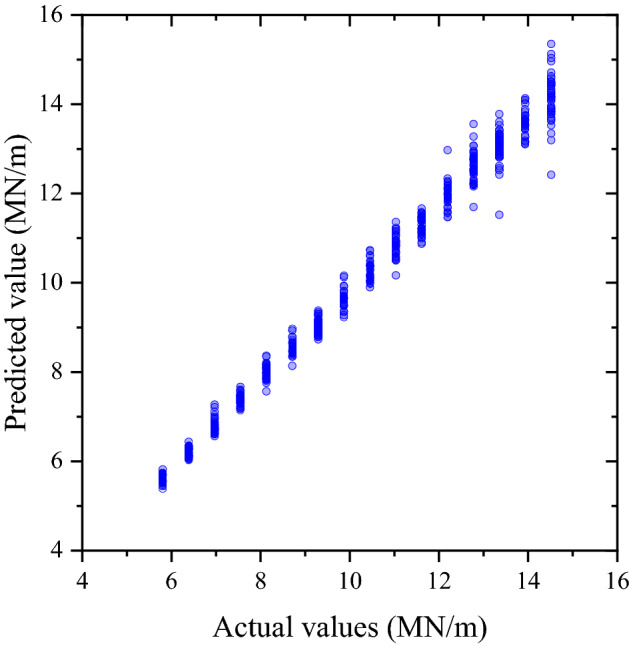
Actual data and prediction.

From the above table, it can be seen that the performances of the regression model conformed to the performance of the classification model. The prediction performance is the best when ABAs and displacement of the crossing nose are used to train the machine learning model followed by the use of ABAs. For the best performance, the MAE is 0.21 MN/m which is relatively low compared to the actual data shown in Table 1. At the same time, the MPE is only 1.95% and the accuracy of the regression model is 98.05%. From the result, it can be concluded that the developed machine learning model has the potential to detect and predict railway turnout support deterioration because of the high accuracy. The actual data and prediction from the regression model can be shown in Fig. 5. The trend is clearly demonstrated in the figure which means the developed machine learning model can do the prediction very well.

### An optimal set of hyperparameters

As mentioned in the methodology, grid search is used to tune hyperparameters. Table 7 presents the optimized combination of tuned hyperparameters.

**Table 7:** Optimized combination of tuned hyperparameters.

Hyperparameters	Values
Number of convolutional layers	2
Filter	64 (conv1) and 32 (conv2)
Kernel size	1
Number of pooling layers	0
Pool size	N/A
Dropout	0.5 (conv2)
Activation function	ReLu
Batch size	64
Optimizer	Adam
Number of hidden layers	2
Number of hidden nodes	100

## Conclusion

This study develops new intelligent automation based on a machine learning pattern recognition model to detect and predict the railway turnout support deterioration which can be used in different situations especially flooding situations. Nonlinear finite element models which are validated by actual field measurements are used to imitate the flooding and train the machine learning models. The key parameter used as a representative of the deterioration is stiffness. The machine learning technique that is used to develop the predictive model is CNN. Data used to train the model is numerical data obtained from LS-DYNA as FEM model simulations. The numerical models are verified with the field measurement to make sure that the outputs from simulations are reliable. Outputs from FEM models that are used to train the machine learning model are ABAs and crossing nose displacement. Outputs from FEM models are used in three forms to investigate the performance of the machine learning model. It is found that the use of both ABAs and crossing nose displacement provides the best performance when the accuracy of the prediction is higher than 0.98 followed by the use of ABAs when the accuracy is higher than 0.97. The use of crossing nose displacement provides provide a lower accuracy which is 0.93. It can be seen that adding one more sensor does not provide a significant improvement in prediction. Therefore, the consideration should be carefully made compared to the cost of additional installation. However, the accuracy of every approach is high. It is to be noted that the features of the machine learning model are in time-series data form. CNN model seems to be well dealing with this format of data because it can detect the pattern hidden in the time-series data. For the deterioration prediction or regression problem, the developed machine learning model provides the highest accuracy of 0.98 when the error is less than 0.21 MN/m which is very accurate and good enough in practice.

The contribution of the study is the developed machine learning model can be used to detect and predict the severity of the railway turnout support deterioration. At the same time, it can be used to predict or estimate the current exact condition of the turnout support. Parties responsible for railway maintenance will be able to improve the railway maintenance plan for better management in terms of time, cost, and quality. The railway operations will be better in the aspect of availability because the severe damages tend to less occur. After all, the maintenance is performed well and the operation will not be disturbed by the heavy damage and long maintenance because the inspection and measurement can be done at the same speed as regular operations and at the same time as regular operations. In addition, the cost of the installation of sensors is relatively cheap compared to other approaches such as the use of specific track measurement cars. In the practice, railway operators can install accelerometers at axle boxes of rolling stocks to measure the ABAs or cameras to measure the displacement of the crossing nose or both. Then, they can use the raw data collected by sensors and feed them into the machine learning model. The machine learning model will detect and predict the deterioration of turnout support deterioration. Then, they can apply the prediction from the machine learning model to the maintenance standard to see the priority of the maintenance. For example, if the deterioration is at the normal level, the maintenance can be done based on the scheduled maintenance plan. If the deterioration is at the priority level, the maintenance needs to be done within the timeframe according to the defined urgent level such as within one month. It can be seen that if the measurement can be done together with regular operations, the data will be collected almost all the time and the deterioration can be tracked and known at every stage of the operation. Therefore, railway operators will know the current conditions of their tracks and respond promptly and properly. This will be a high benefit in terms of railway maintenance because the cost can be managed more efficiently and the availability of the whole system can be improved.

## Data Availability

The data that support the findings of this study are available from Brazilian Railway Authority but restrictions apply to the availability of these data, which were used under license for the current study, and so are not publicly available. Data are however available from the authors upon reasonable request and with permission of the Brazilian Railway Authority.
